# Taxonomic validation of Ephoron yunnanensis Zhou, 2002 (nomen nudum) (Ephemeroptera, Polymitarcyidae)

**DOI:** 10.3897/zookeys.1286.204617

**Published:** 2026-07-27

**Authors:** Sedtawut Kwanboon, Xu-Hong-Yi Zheng, Chang-Fa Zhou, Boonsatien Boonsoong

**Affiliations:** 1 Animal Systematics and Ecology Speciality Research Unit (ASESRU), Department of Zoology, Faculty of Science, Kasetsart University, Bangkok 10900, Thailand The Key Laboratory of Jiangsu Biodiversity and Biotechnology, College of Life Sciences, Nanjing Normal University Nanjing China; 2 The Key Laboratory of Jiangsu Biodiversity and Biotechnology, College of Life Sciences, Nanjing Normal University, Nanjing 210023, China Animal Systematics and Ecology Speciality Research Unit (ASESRU), Department of Zoology, Faculty of Science, Kasetsart University Bangkok Thailand; 3 Biodiversity Center Kasetsart University (BDCKU), Bangkok, Thailand Biodiversity Center Kasetsart University (BDCKU) Bangkok Thailand

**Keywords:** Burrowing mayfly, COI, egg, taxonomy

## Abstract

Ephoron yunnanensis Zhou, 2002 is considered as a nomen nudum since only mentioned in an unpublished Ph.D. thesis and therefore not officially validated. This study validates the species based on specimens from China (Yunnan Province) and Thailand (Chiang Mai and Chiang Rai Provinces). The species is formally described here for the first time and illustrated. Our morphological evidence and COI data support its independent species status. Additionally, the egg of this species is described. Diagnostic characters, distributions, and keys for both the nymphal and imaginal stages of all known Thai Ephoron species are also provided.

## Introduction

The genus Ephoron Williamson, 1802 (Ephemeroptera, Polymitarcyidae) is widely distributed throughout the Nearctic, Palaearctic, Oriental, and Afrotropical regions. This genus is commonly found in rivers and other lowland water bodies (Ishiwata 1996). The nymphal stage exhibits filter-feeding behaviour; it burrows into the riverbed and feeds on small particles in the current, which are carried by the movement of its abdominal gills (Cid et al. 2008). Male imago mate with female subimago and have a very short lifespan (Edmunds and McCafferty 1988). Therefore, the imaginal stage of this genus is often characterised by mass swarms in certain localities (Edmunds et al. 1976; Ishiwata 1996; Tojo et al. 2006; Száz et al. 2015).

Ephoron yunnanensis was first named by Zhou in his unpublished doctoral dissertation in 2002. Since then, this species has been mentioned in various publications by Chinese researchers, for example, by Yu et al. (2021), who included this species in their tables 1, 2 and fig. 3. In addition, Zhou et al. (2003) also cited this species. However, because no formal description of *E. yunnanensis* has ever been published, it is a nomen nudum under the International Code of Zoological Nomenclature (ICZN 1999). As a result, its taxonomic status remained uncertain for decades.

In Thailand, three species of the genus Ephoron have been recognised, namely *E. indicus* (Pictet, 1843), *E. ookaewae* Techakijvej & Phalaraksh, 2021, and *E. debaratana* Kwanboon, Auychinda, Suttinun & Boonsoong, 2025 (Uéno 1961; Techakijvej et al. 2021; Kwanboon et al. 2025). Recent surveys of mayfly diversity in northern Thailand allow to collect imaginal and nymphal stages of the genus Ephoron that could not be assigned to any known species. These specimens closely matched the species from Yunnan Province, prompting a re-evaluation of *E. yunnanensis*. Thus, the nomen nudum Ephoron yunnanensis is validated here, and mitochondrial COI sequences are used to further support this species identity.

## Materials and methods

### Specimen collecting

The nymphs were collected by a D-framed net in rivers from China and Thailand. The imagoes were collected by the light trap method at night near the river. All specimens were preserved in absolute ethanol.

### Morphology observation

Measurements (in mm) and photographs were taken using a stereomicroscope (Nikon SMZ800 and ZEISS Stemi 305). For scanning electron microscopy (SEM), specimens of egg, first instar nymphal stage and genitalia were dried in a critical point dryer (CPD7501) and coated with gold (Sputter Coater SC7620). The specimens were observed and photographed with a FEI Quanta 450 SEM. The final plates were prepared with Adobe Photoshop CC 2022. The distribution map was constructed using the Simple Mapper website (http://www.simplemappr.net) (Shorthouse 2010) and GPS coordinates. The material was deposited in the Mayfly Collection, College of Life Sciences, Nanjing Normal University (**NNU**) and the collection of the Zoological Museum at Kasetsart University in Bangkok, Thailand (**ZMKU**).

### Molecular analysis

Selected specimens were dissected for DNA extraction. Total DNA was extracted using a genomic DNA purification kit (NucleoSpin, Macherey-Nagel, Germany) according to the manufacturer’s protocol. The COI gene amplification was performed using LCO1490 and HCO2198 (Folmer et al. 1994). The polymerase chain reaction (PCR) conditions and procedure were as described by Kwanboon et al. (2021). The PCR products were sent to U2Bio Co., Ltd (Korea) for purification and sequencing. The genetic distances between the species were determined using Kimura 2-parameter (K2P) distances (Kimura 1980), calculated using the MEGA11 program (Tamura et al. 2021). Sequence alignment and editing were performed using ClustalW in MEGA11. A phylogenetic tree was analysed using the maximum-likelihood (ML) method, and the most appropriate evolutionary model was calculated using the Find Best DNA/Protein Models (ML) option test provided with MEGA11. The general time-reversible (GTR) nucleotide substitution model with a gamma distribution for rate variation across sites (+G) and a proportion of invariable sites (+I) was prepared using MEGA11 and the likelihood-ratchet method with 1000 bootstrap replicates. The nucleotide sequences obtained in this study were deposited in GenBank.

## Taxonomy

**Order Ephemeroptera Hyatt & Arms, 1891**

**Family Polymitarcyidae Banks, 1900**

**Genus Ephoron Williamson, 1802**

### Ephoron yunnanensis

Taxon classificationAnimaliaEphemeropteraPolymitarcyidae

Zhou sp. nov.

F1D31B7E-0485-53BB-ABB3-D7252C5C78D7

https://zoobank.org/2BA7643D-1D08-46BE-9187-C09C8397234B

Figs 1, 2, 3, 4, 5, 6, 7, 8, 9, 10, 11, 12, 13, 14, 15, 16, 17, 18, 19, 20

Ephoron yunnanensis Zhou, 2002 (nomen nudum) (in doctoral dissertation).

#### Material examined.

***Holotype***: China • 1 male imago, Yunnan Province, Jinggu County, Fengshan village; 23°11'54.10"N, 100°8'24.75"E; 8.IV.2001, ZHOU Chang-Fa leg. (NJNU Mayfly2607171). ***Paratypes***: China • 3 male imagos, 10 female subimagos, 5 nymphs, same data as for holotype (NJNU Mayfly2607172).

#### Other materials.

China • 2 male imagos, Yunnan Province, Xishuangbanna Dai Autonomous Prefecture, Menglun County, Nanban River, 21°56'4.67"N, 101°15'3.36"E, 542 m a.s.l., 25. I. 2023, Xuhongyi Zheng leg. (NJNU Mayfly2607173). Thailand • 105 subimagoes, 12 nymphs, Chiang Mai Province, Chang Khoeng, Mae Chaem River, 470 m a.s.l., 18°30'46"N, 98°21'24"E, 15.XII.2024, B. Boonsoong, S. Kwanboon, C. Auychinda leg. (ZMKU Ephe-048). THAILAND • 7 nymphs, Chiang Rai Province, Mae Kok River, 398 m a.s.l., 19°55'50.3"N, 99°46'19.0"E, 01.IX.2019, B. Boonsoong, S. Kwanboon, C. Auychinda leg. (ZMKU Ephe-049).

#### Diagnosis.

**Figure 1.**
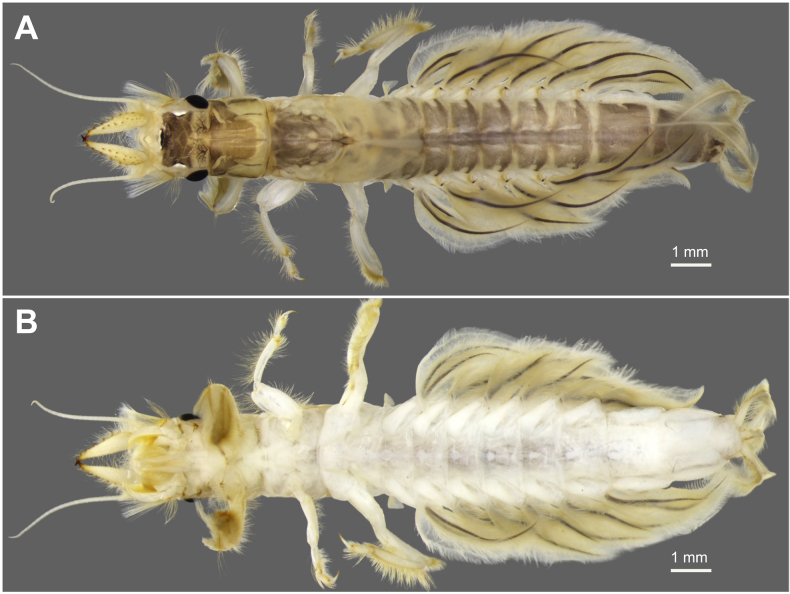
Habitus of Ephoron yunnanensis sp. nov. nymph. **A**. Dorsal view; **B**. Ventral view.

The nymphs of Ephoron yunnanensis sp. nov. can be separated from those of other Ephoron species based on the following characteristics: i) blunt small spine between outer and inner incisors on right mandible (Fig. 3B), ii) frontal process triangular (Fig. 1A), iii) abdominal gill without lateral pigmented branch (Fig. 6B). In male imago, six intercalary veins in CuA field of forewing (Fig. 6A), colour pattern of abdominal terga (Fig. 7A), five, four, and four tarsomeres in foreleg, midleg, and hindleg respectively; moderately deep median cleft of penis (Fig. 10A, B). In female subimago, legs poorly developed, with forelegs 4-segmented, other legs 3-segmented, and egg oval with a smooth chorion (Fig. 14A, C).

#### Description.

***Nymph* (*in alcohol*)**: body length 9.7–15 mm (*n* = 5), caudal filaments shorter than body length (Fig. 1A, B). **Coloration**. ***Head***. Brownish, dark brownish between ocelli. Antenna pale yellowish. Compound eye black, ocelli white, inner margin of ocelli black. Mouthparts pale brown. ***Thorax***. Brown as in Fig. 1A. Legs whitish to yellowish. ***Abdomen***. Brown, with dark brown markings along the terga. Sterna whitish. Gills whitish to yellow. Caudal filament uniformly yellowish.

**Head**. Prognathous, trapezoidal in dorsal view, anterior margin with a triangular frontal process (Fig. 1A).

**Figure 2.**
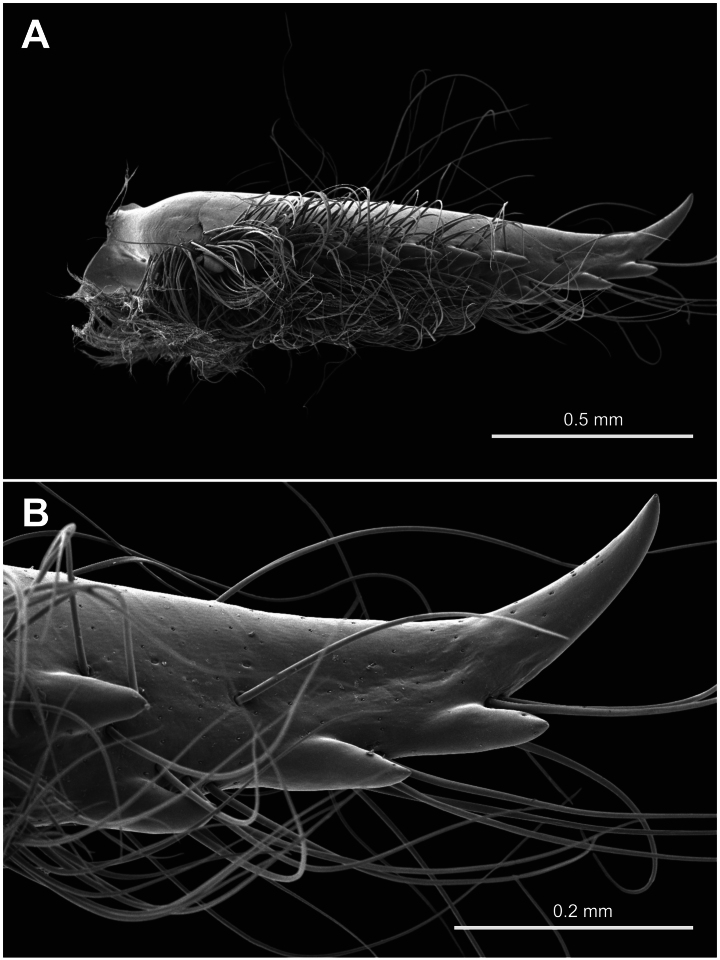
Ephoron yunnanensis sp. nov., nymphal morphology. **A**. Right mandibular tusk (lateral view); **B**. Apex and tubercles of right mandibular tusk (lateral view).

**Figure 3.**
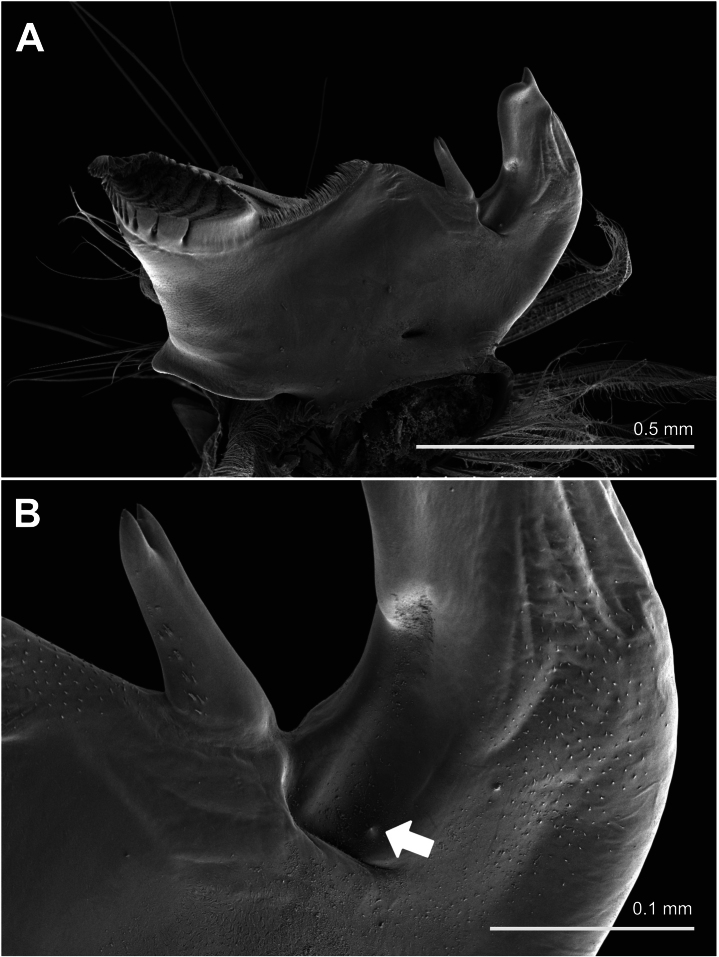
Ephoron yunnanensis sp. nov., nymphal morphology. **A**. Right mandible (anteroventral view); **B**. Spine (arrow) between outer and inner incisors of right mandible.

**Figure 4.**
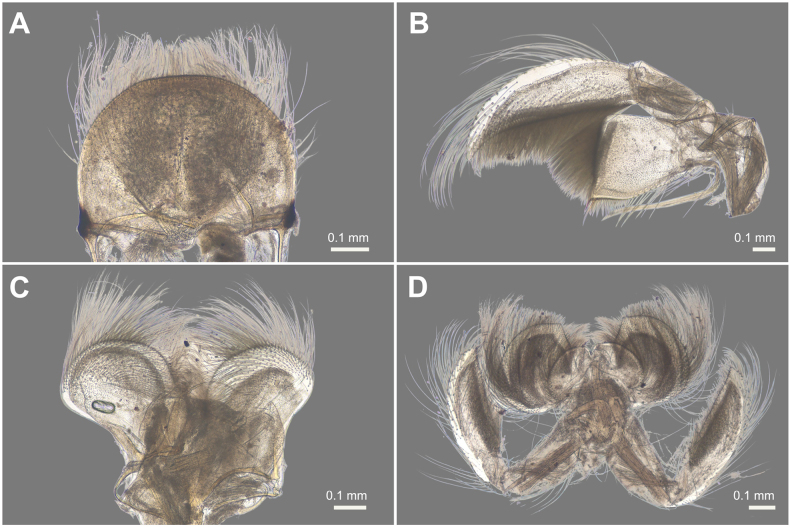
Ephoron yunnanensis sp. nov., nymphal morphology. **A**. Labrum (dorsal view); **B**. Left maxilla (ventral view); **C**. Hypopharynx (ventral view); **D**. Labium (ventral view).

**Mouthpart. *Labrum*** rounded, ca 1.5 times wider than long, covered with numerous hair-like setae in dorsal surface (Fig. 4A). ***Mandibular tusk*** yellowish to golden, apex brown and slightly curve inward; tusk cover with dense setae in dorsal base, over 17 brownish tubercles on dorsal and lateral margin (Fig. 2A, B); only right mandible with small, reduced spine between outer and inner incisor (Fig. 3A, B). ***Labium*** covered with dense setae, paraglossae and glossae drop shape with numerous setae, labial palpi 2-segmented, segment I shorter than segment II, both covered with setae (Fig. 4D). ***Maxilla*** stout, with relatively broad, subrectangular, crown covered with dense setae, distal margin simple without denticles; maxillary palpi 2-segmented, first segment shorter than second segment, second segment similar in shape to the second segment of labial palp (Fig. 4B, D). ***Hypopharynx*** broad, with well-developed superlinguae; each superlingua rounded and densely covered with long, hair-like setae. Lingua narrow, distinctly shorter than superlinguae, apical margin rounded (Fig. 4C).

**Figure 5.**
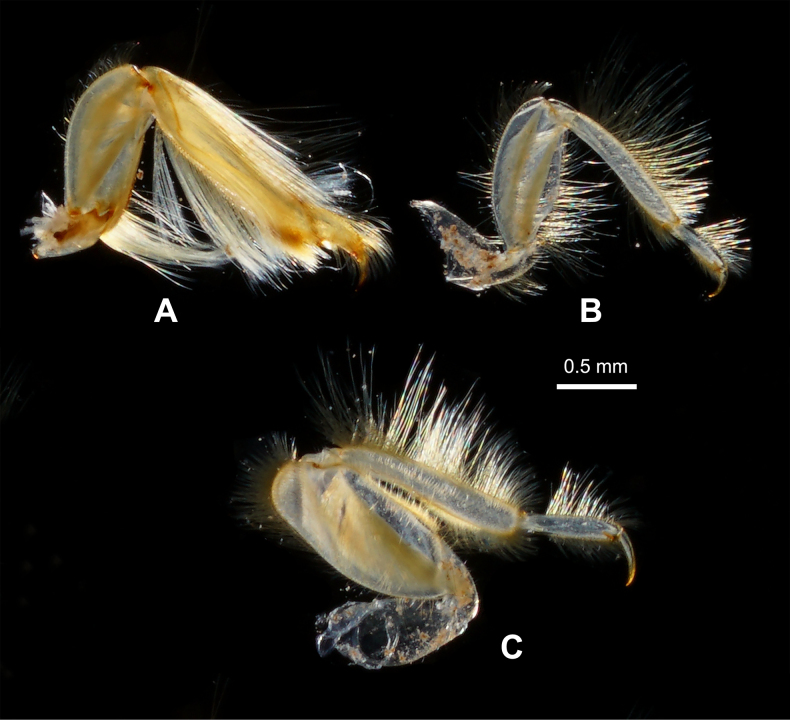
Ephoron yunnanensis sp. nov., nymphal morphology. **A**. Foreleg; **B**. Middle leg; **C**. Hind leg.

**Thorax**. Dorsal and ventral surfaces covered with long, fine, yellowish setae. Forelegs strongest, stout and fossorial with several distinct rows of long simple setae; ventral margin of femora with a basal row of tubercles; tibiae flattened, inner margin with two rows of tubercles; tarsal claws hooked and stout (Fig. 5A–C), tibia and tarsus much wider than in midleg and hindleg, length ratio of femur: tibia: tarsus ca 1.0: 1.3: 0.3. Midleg shortest, similar in structure to foreleg, length ratio of femur: tibia: tarsus ca 1.0: 1.1: 0.4. Hindleg similar in structure to foreleg, length ratio of femur: tibia: tarsus ca 1.0: 1.2: 0.5.

**Figure 6.**
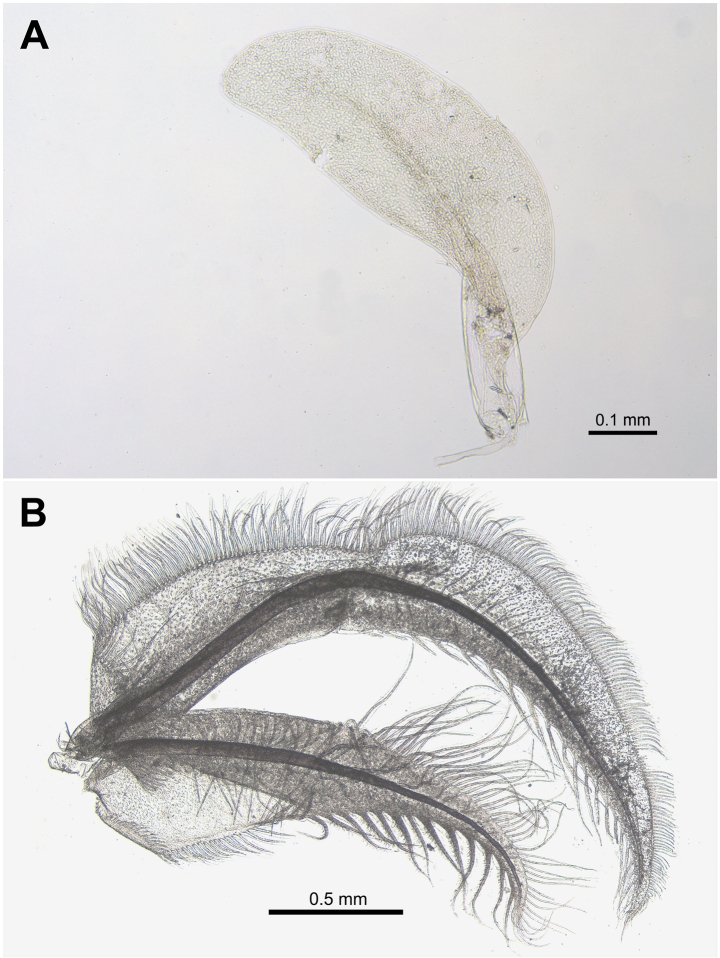
Ephoron yunnanensis sp. nov., nymphal morphology. **A**. Gill I; **B**. Gill III.

**Abdomen**. gills on abdominal segments I–VII, with black dot at base of gills II–VII, gill I uniramous, banana-shaped (Fig. 6A); gills II–VII similar in shape, biramous, both lamellae fringed with hair-like setae, dorsal lamella slender and slightly curved, outer margin with a shallow depression medially, expanded basally, without lateral pigmented branch (Fig. 6B). Cerci and paracercus subequal in length, with setae.

**Figure 7.**
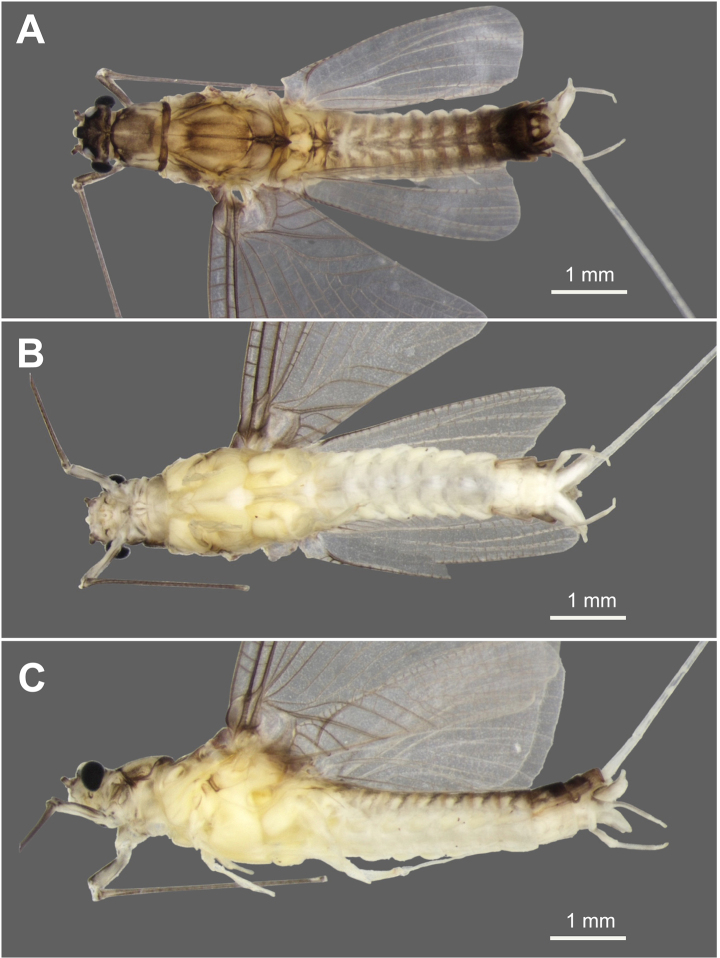
Ephoron yunnanensis sp. nov., male imago morphology. **A**. Dorsal view; **B**. Ventral view; **C**. Lateral view.

***Male imago* (*in alcohol*)**: body length 10–13 mm (*n* = 5). caudal filaments longer than body length**. Colouration**. ***Head***. yellowish to brownish, dark brown between ocelli. Antenna whitish, darker at base. Compound eye black, ocelli white, inner margin of ocelli black. ***Thorax***. Yellowish with brown markings (Fig. 7A). ***Abdomen***. Abdominal terga whitish, with dark-brownish markings becoming darker and wider posteriorly, and darkest on segments VIII–X (Fig. 7A, C). Abdominal sterna whitish without markings (Fig. 7B).

**Head**. Head width slightly less than pronotum width (Fig. 7A). Compound eye globular on posterolateral margin of head.

**Figure 8.**
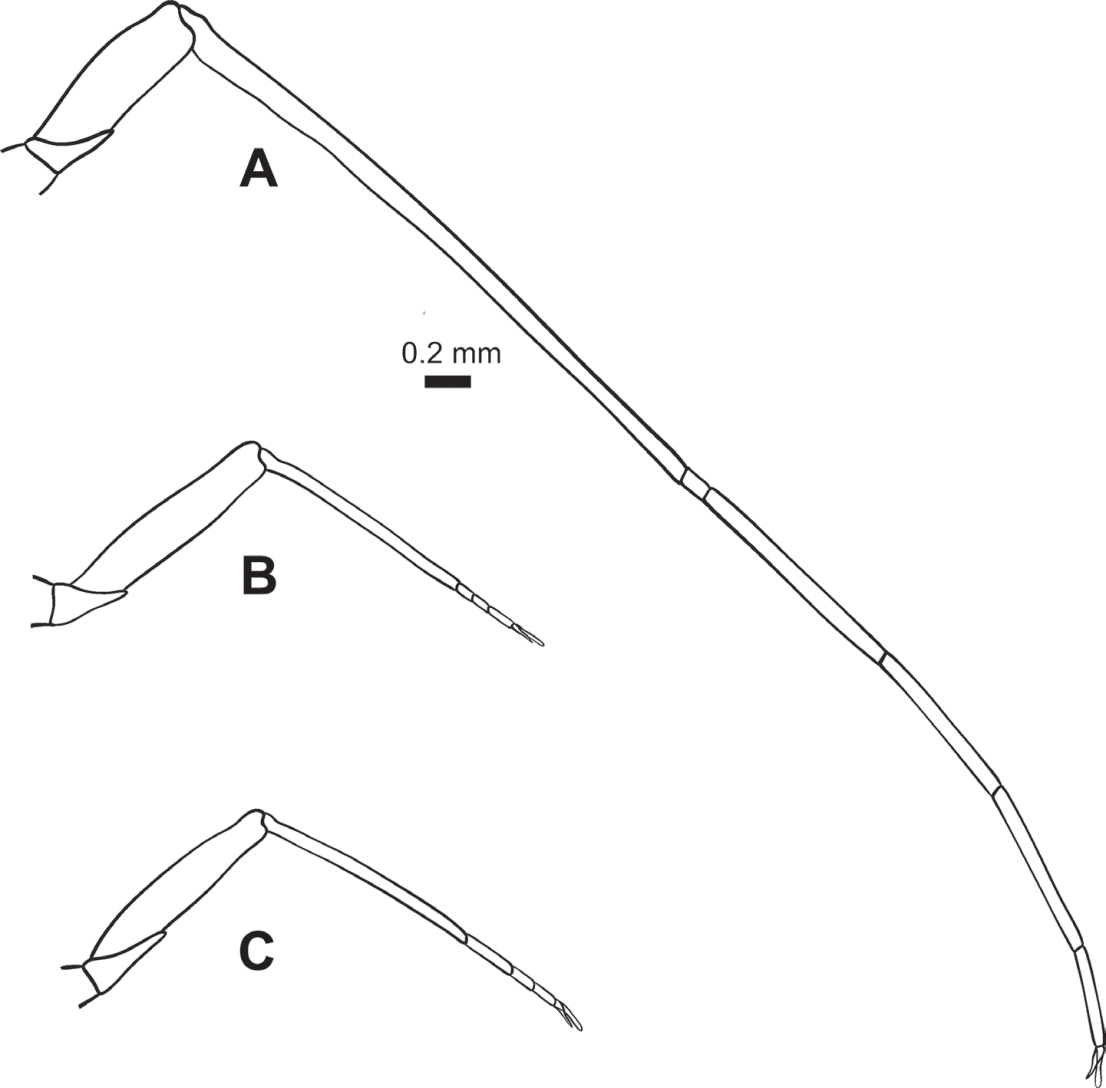
Ephoron yunnanensis sp. nov., male imago morphology. **A**. Foreleg; **B**. Middle leg; **C**. Hind leg.

**Figure 9.**
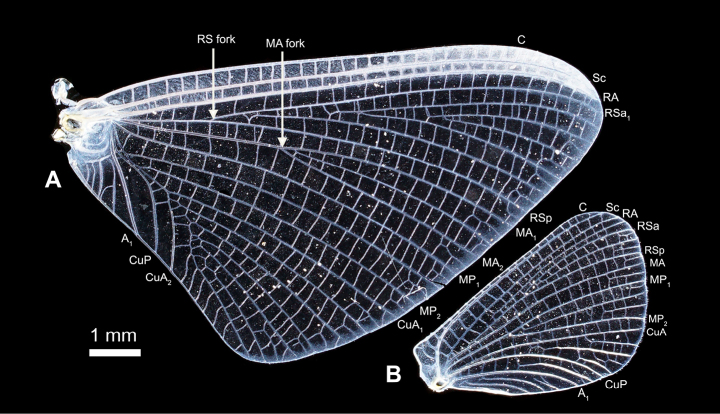
Ephoron yunnanensis sp. nov., male imago morphology. **A**. Forewing; **B**. Hindwing.

**Thorax. Foreleg** well developed and slender, five segments of tarsomeres, length ratio of femur, tibia and five tarsomeres ca 1: 4: 0.2 :1.3 :1.0 :1.0 :0.6, claws paired, rounded apically. Midleg and hindleg greatly reduced and much shorter than foreleg (Fig. 8A–C). **Midleg** shortest, length ratio of femur, tibia, and tarsus ca 1: 1.1: 0.4, tarsus with paired claw, length ratio of tarsomeres ca 0.1: 0.1: 0.2. **Hindleg** similar midleg in shape but longer, length ratio of femur, tibia, and tarsus ca 1: 1.2: 0.4, length ratio of tarsomeres ca 0.2: 0.1: 0.1. **Forewings** hyaline ca 10.5 mm in length, costal margin slightly brownish, apical and cubital angle rounded, numerous cross-veins (Fig. 9A). Fork of MA distal to fork of RS, 1 intercalary between MA1 and MA2. RS forked at basal 1/4 with five intercalaries in RS fork. MP asymmetrical forked at base. CuA forked, with six intercalaries between CuA1 and CuA2; CuP and CuA2 parallel; A1 straight (Fig. 9A). **Hindwings** ca 0.5× forewing in length, hyaline, with a blunt costal-basal projection; one intercalary between RA and RSa, RS fork at middle of wing; MA single, MP fork symmetrically near 1/3 of base wing; 3 intercalaries between CuA and CuP (Fig. 9B).

**Figure 10.**
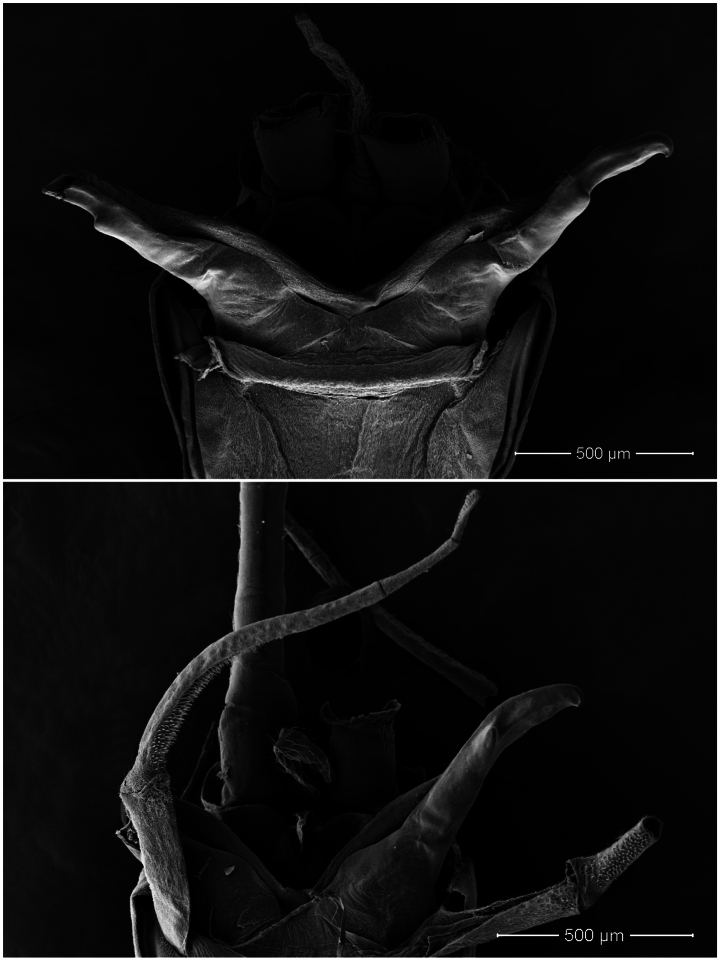
Ephoron yunnanensis sp. nov., male genitalia morphology. **A**. Ventral view; **B**. Dorsal view.

**Abdomen**. Genitalia with four elongated segmented forceps, first and second segments similar in length, third segment longest. Penis diverging laterally and hook and the apex of each lobe, subapical margin distinctly concave, apices rounded, moderately deep median cleft (Fig. 10A, B).

***Female subimago* (*in alcohol*)**: Body length 12.8–15.3 mm (*n* = 5) excluding caudal filament and cerci. Sexually mature in this stage.

**Figure 11.**
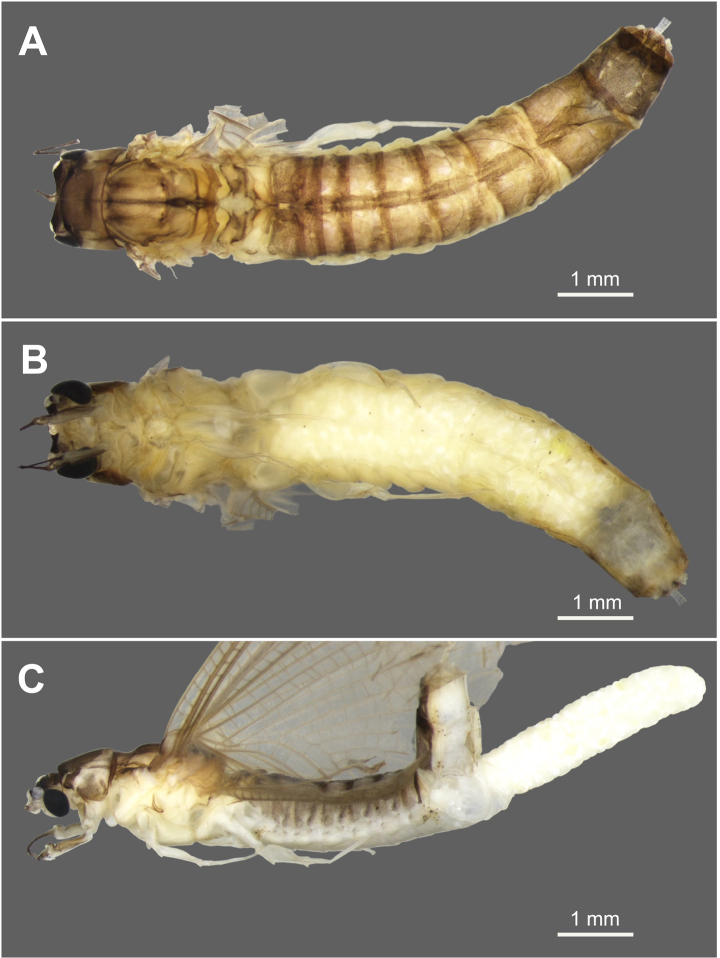
Ephoron yunnanensis sp. nov., female subimago morphology. **A**. Dorsal view; **B**. Ventral view; **C**. Lateral view.

**Colouration**. ***Head***. Markings and colour similar to male (Fig. 11A). ***Thorax***. Colour and pattern same as male. ***Abdomen***. Abdominal terga whitish, with dark-brownish markings becoming darker and wider posteriorly, and darkest on segments VIII–X (Fig. 11A–C). Abdominal sterna whitish without markings (Fig. 11B).

**Figure 12.**
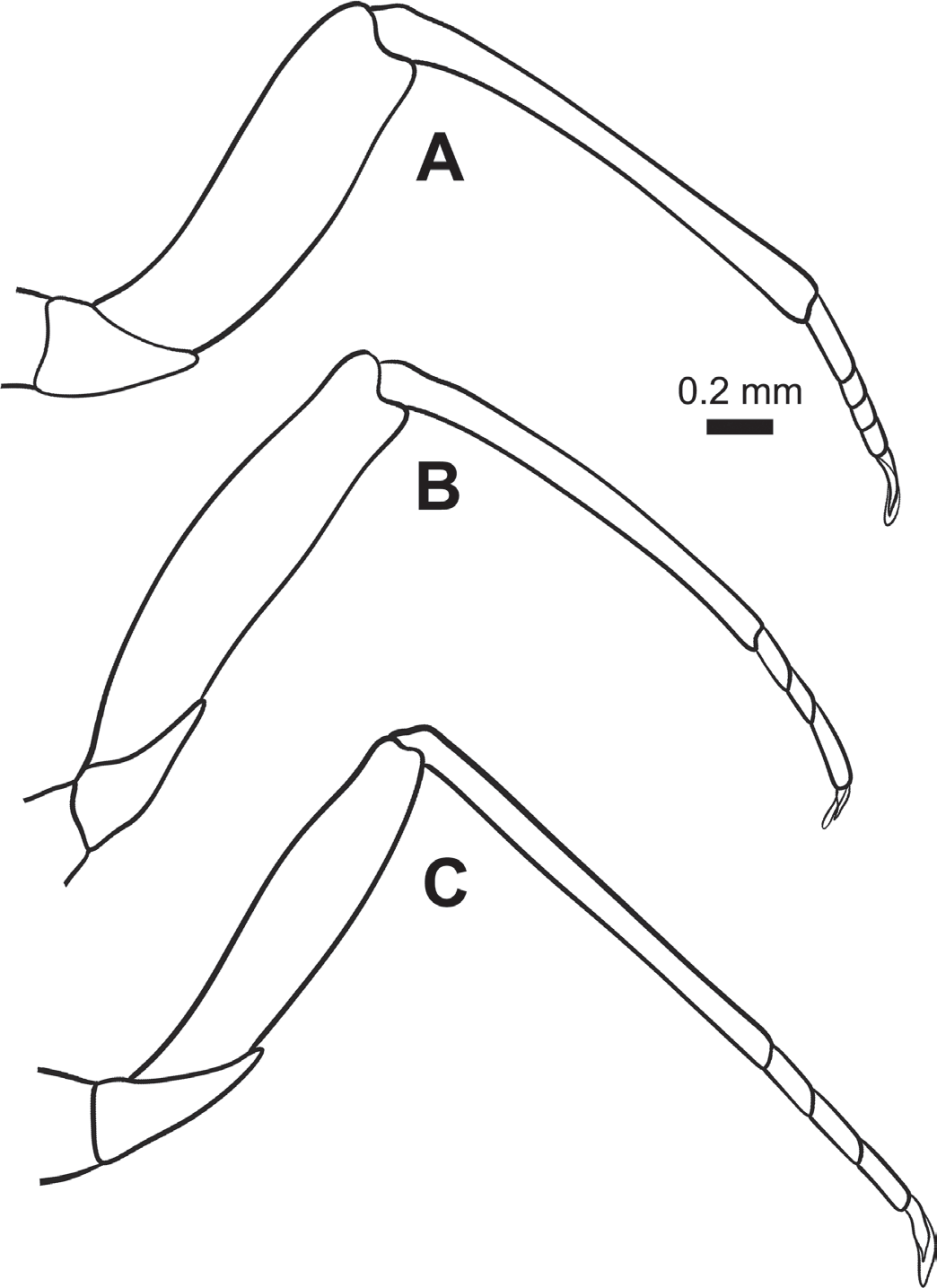
Ephoron yunnanensis sp. nov., female subimago morphology. **A**. Foreleg; **B**. Middle leg; **C**. Hind leg.

**Figure 13.**
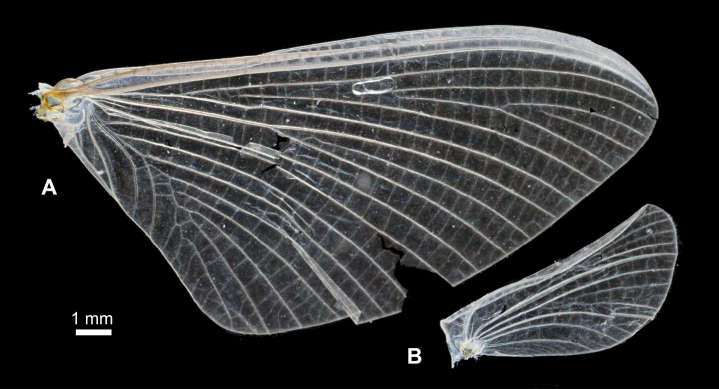
Ephoron yunnanensis sp. nov., female subimago morphology. **A**. Forewing; **B**. Hindwing.

**Thorax. Foreleg** poorly developed, four segments of tarsomeres, length ratio of femur, tibia tarsus and four tarsomeres ca 1.0: 1.2: 0.4: 0.1: 0.1: 0.1, claws paired, rounded apically (Fig. 12A). **Midleg** shortest, femur: tibia: tarsus ratio 1.0: 1.1: 0.4, three segments of tarsomeres, length ratio ca 0.1: 0.1: 0.2 (Fig. 12B)**. Hindleg** slightly longer than midleg, femur: tibia: tarsus ratio 1.0: 1.0: 0.3, tarsus three segmented, length ratio ca 0.1: 0.1: 0.1 (Fig. 12C). **Forewings** ca 19 mm in length, translucent, costal margin slightly brownish, overall shape and venation comparable to male, except for the presence of five intercalary veins between CuA1 and CuA2 (Fig. 13A). **Hindwings** translucent, costal projection well developed, venation similar to male (Fig. 13B).

**Abdomen**. Cerci and paracercus subequal in length, shorter than body length, covered with setae.

**Figure 14.**
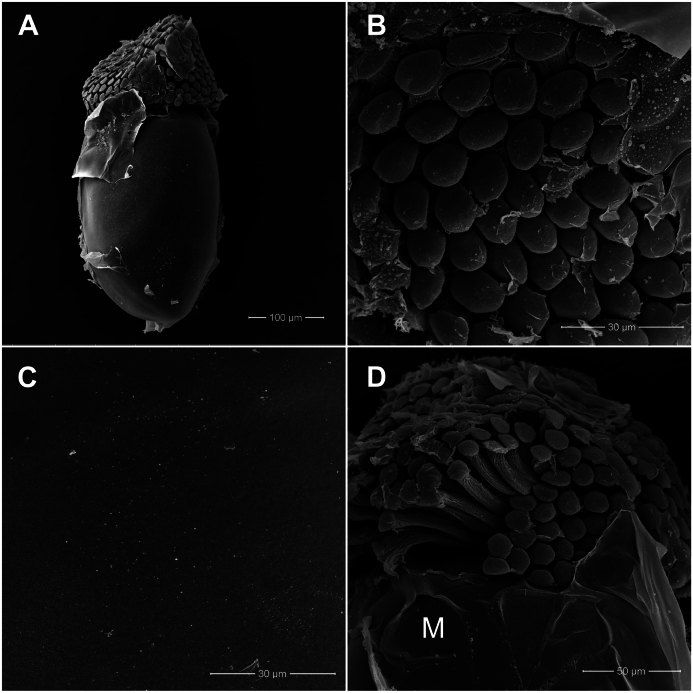
Ephoron yunnanensis sp. nov., SEMs of egg structures. **A**. General outline of egg; **B**. Polar cap; **C**. Chorion surface; **D**. Micropyle.

**Egg**. Dissected from female subimago; length 282–328 μm, width 183–224 μm (*n* = 5); Elongate-oval, with one large polar cap (Fig. 14A, B); polar cap irregular in outline, occupying ca 1/3 of egg length, its margin distinctly separated from main chorion; chorion smooth, without any reticulation (Fig. 14C); opposite pole slightly tapering, without a concave depression; one visible micropyle situated beneath polar cap (Fig. 14D). Eggs tightly grouped together form two elongate-oval, yellow egg masses, hard in air, sticky after contact with water (Figs 11C, 18D).

#### Description of the first instar.

**Figure 15.**
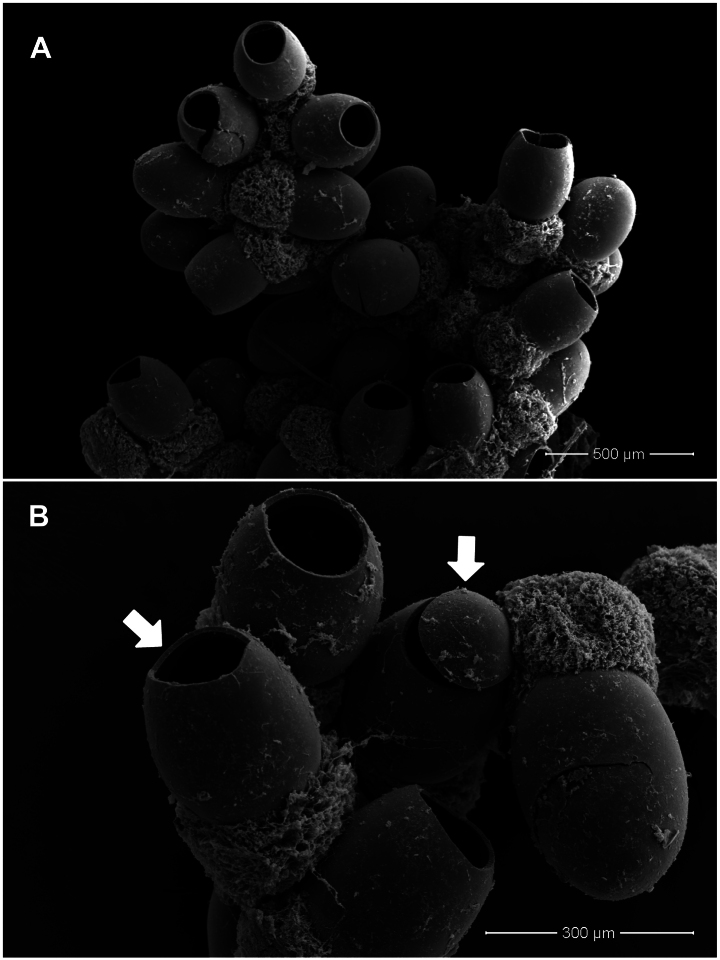
SEMs of egg mass of Ephoron yunnanensis sp. nov. **A**. General outline of hatched and unhatched eggs; **B**. Enlargement of egg chorion at uncapped pole (arrow) of hatched egg.

**Figure 16.**
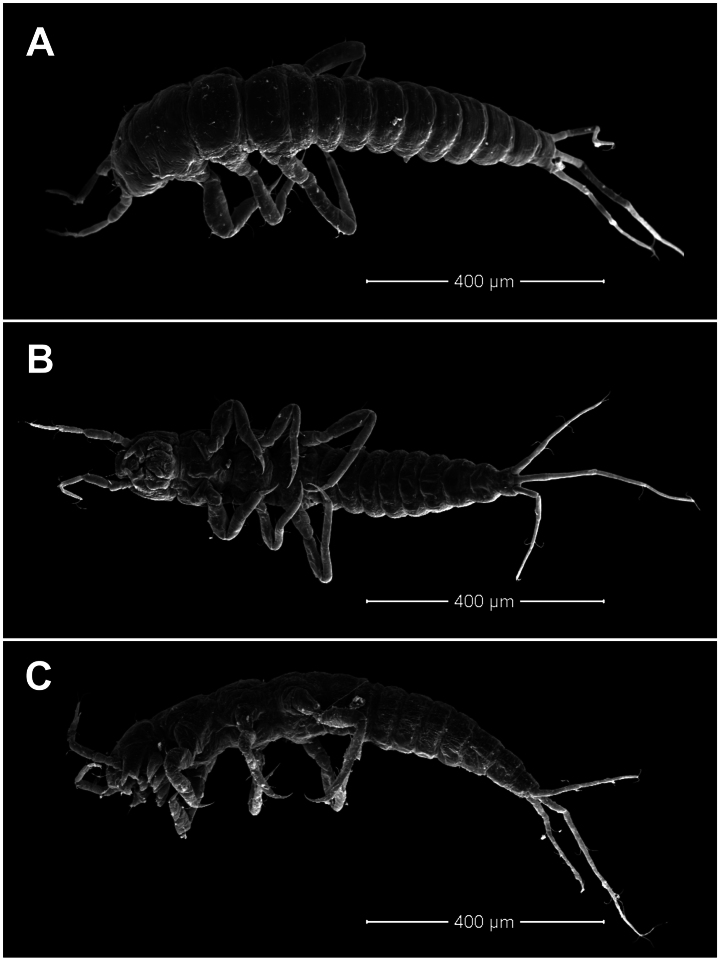
SEMs of habitus of the first instar of Ephoron yunnanensis sp. nov. **A**. Dorsal view; **B**. Ventral view; **C**. Lateral view.

**Figure 17.**
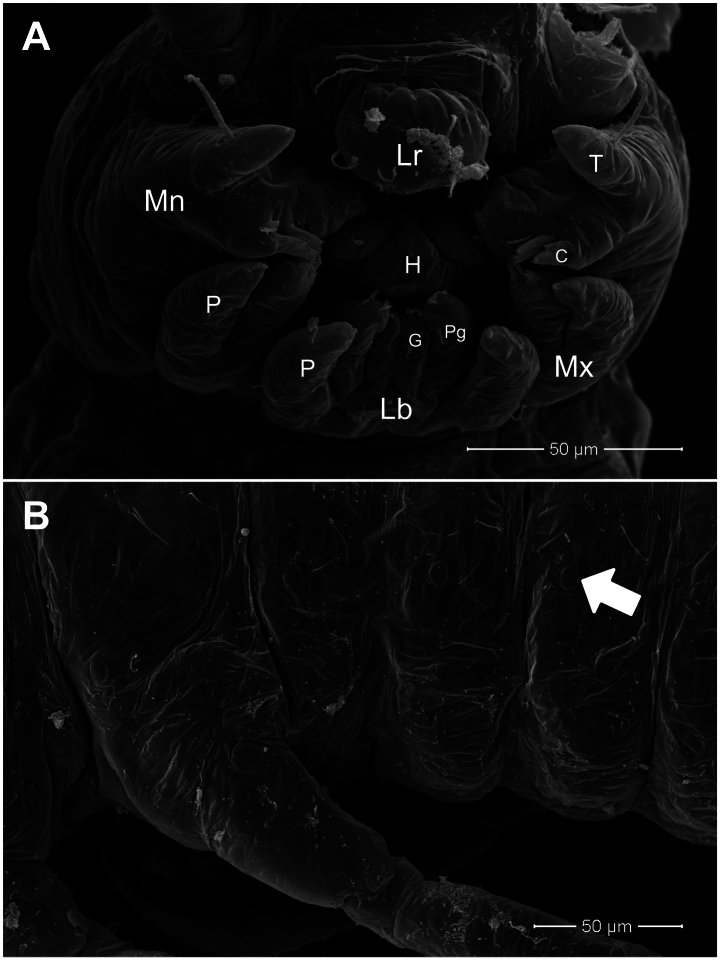
SEMs of the first instar of Ephoron yunnanensis sp. nov. **A**. Mouthpart (ventral view); **B**. Metathorax and abdomen (lateral view) (arrow indicated spiracle). Abbreviation: Lr (Labrum), Mn (Mandible), Mx (Maxilla), Lb (Labium), P (Palp), T (tusk), Pg (paraglossae), G (glossa), C (canine region), H (hypopharynx).

Egg masses of *E. yunnanensis* were immersed in water under laboratory conditions; polar caps of egg facilitated attachment to substrate (Fig. 15A). Embryonic development complete and hatching after 12 days. *E. yunnanensis* hatches from its egg case at the uncapped pole, the chorion opens in a circular manner as in Fig. 15B. Body length of first instar 560–680 μm (Fig. 16A–C). ***Mouthparts***. Mandibles of first instar large compared to head capsule size; in distal region of mandible, mandibular tusk begins to develop, located perpendicular (ca 90°) to canine region, apex pointing toward canine region; distal-most tips of mandibular tusks modified into small teeth in canine region (Fig. 17A). Maxillae bare, with small tooth structure; galea lacinia fused; palps unsegmented (Fig. 17A). Labium with short palp, with four lobes between palp corresponding to two outer paraglossae and two inner glossae (Fig. 17A). Labrum rounded, with four or five short setae (Fig. 17A). ***Thorax***. All legs present: coxae, trochanters, femora, tibiae, tarsi, and claws already differentiated. Legs sparsely setose (Fig. 16)**. *Abdomen***. Gill absent (Fig. 17B). Three caudal filaments present.

#### Etymology.

The specific epithet *yunnanensis* refers to Yunnan Province, where the type locality is located.

#### Biology.

**Figure 18.**
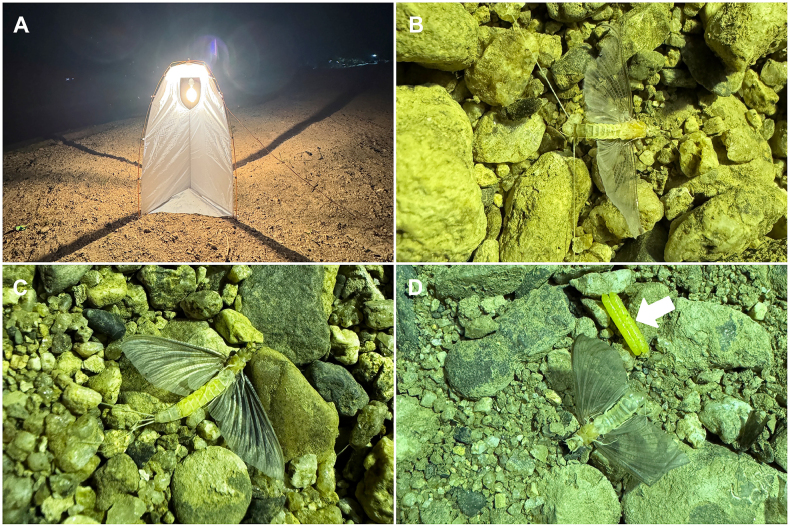
Light trap near the Mae Chaem wadeable river (March 2025). **A**. Light trap; **B**. Male imago; **C**. Female subimago; **D**. Female subimago with egg masses (arrow indicates egg mass).

In Thailand, the mass emergence of *E. yunnanensis* typically began around 19.00 and finished approximately at 20.30 (GMT +7). Both sexes were attracted by a light trap set near the Mae Chaem River, as observed on 29 March 2025 (Fig. 18A). Among the emerging individuals, mostly female subimagoes were found (Fig. 17B, D). The sex ratio (female to male) was 2:1. The female subimagoes were observed fluttering their wings over the rocky substrate adjacent to the streambank, attempting to oviposit (Fig. 17C, D). The egg bar appeared as a yellowish rod-like structure (Fig. 17D). The female typically dies shortly after oviposition. However, no mating behaviour between males and females was observed during this survey.

#### Distribution.

China (Yunnan Province) and Thailand (Chaing Mai and Chiang Rai Provinces) (Fig. 20).

#### Molecular analysis.

The partial sequences of the mitochondrial COI gene (658 bp) of the *E. yunnanensis* sp. nov. from China and Thailand were obtained from specimens of each locality. In addition, other Ephoron species were combined to investigate species delineation (Table 1). The K2P analysis revealed that the mean intraspecific genetic distance among Thai and Chinese populations of *E. yunnanensis* sp. nov. is 2.52%, and for *E. debaratana* 2.88%. The mean interspecific distances between Ephoron are high, ranging from 4.60% to 26.84% (Table 2). The interspecific genetic distances among the Thai species of Ephoron ranged from 20.37% to 22.10%. Pairwise genetic distances were 20.37% between *E. yunnanensis* sp. nov. and *E. ookaewae*, 20.78% between *E. debaratana* and *E. ookaewae*, and 22.10% between *E. yunnanensis* sp. nov. and *E. debaratana*.

**Table 1.** Sequenced specimens of the genus Ephoron in this study.

**Species**	**Locality**	**GenBank accession no. / code**
*E. debaratana*	Kanchanaburi, Thailand	PV920440
	Kanchanaburi, Thailand	PV920440
*E. yunnanensis* sp. nov.	Chiang Rai, Thailand	PZ683037 / EP05CR
	Chiang Rai, Thailand	PZ683038 / EP06CR
	Chiang Mai, Thailand	PZ683039 / EP07CM
	Yunnan, China	PZ683040 / EP_China
*E. ookaewae*	Nakhon Sawan, Thailand	MW168821.1
*E. nanchangi*	China	OQ439818
*E. shigae*	Japan	LC635033
	Japan	LC635042
*E. limnobium*	Japan	AB711781
	Japan	AB711785
*E. eophilum*	Japan	AB711807
	Japan	AB711808
*E. virgo*	Europe	PP402759.1
	Europe	PP403796.1
*E. nigridorsum*	Mongolia	LC102558.1
	Mongolia	LC102557.1

**Table 2.** Genetic distances (COI) between sequenced specimens using the Kimura 2-parameter method.

	**Species**	**1**	**2**	**3**	**4**	**5**	**6**	**7**	**8**	**9**
**1**	*E. nanchangi*									
**2**	*E. debaratana*.	21.67								
**3**	*E. yunnanensis* sp. nov.	23.23	22.10							
**4**	*E. ookaewae*	23.70	20.78	20.37						
**5**	*E. shigae*	21.05	18.77	23.23	23.22					
**6**	*E. limnobium*	20.93	19.32	22.42	22.63	4.60				
**7**	*E. eophilum*	23.31	16.24	20.04	22.10	16.19	17.11			
**8**	*E. virgo*	13.35	19.00	18.66	21.66	18.33	17.39	16.63		
**9**	*E. nigridorsum*	26.84	23.38	22.53	26.81	23.22	24.41	22.69	24.28	

**Figure 19.**
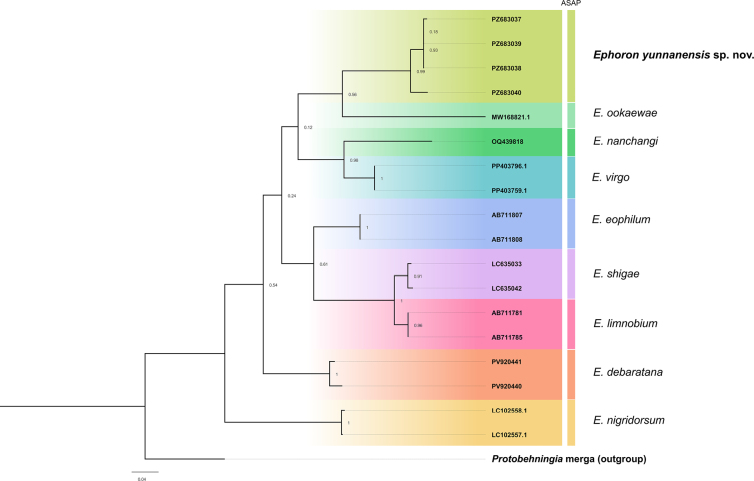
COI phylogenetic construction based on the maximum-likelihood analysis of nine Ephoron species. Number at the node indicates bootstrap values.

The maximum-likelihood (ML) analysis of COI supports the monophyly of three Thai species (Fig. 19). The newly obtained sequences of *E. yunnanensis* sp. nov. formed a distinct and well-supported monophyletic clade, clearly separated from its congeners. Within *E. yunnanensis* sp. nov., the Thai and Chinese specimens clustered together with strong nodal support. Ephoron yunnanensis sp. nov. was inferred as a sister lineage to *E. ookaewae*, while *E. debaratana* formed a separate distant clade, separated from the other Thai species. Additionally, the Japanese species; *E. eophilum*, *E. shigae*, and *E. limnobium* formed a distinct monophyletic clade. The ASAP species delimitation analysis identified nine molecular clusters, with *E. yunnanensis* sp. nov. recovered as a distinct operational taxonomic unit (OTU) separate from all other congeners, consistent with the ML topology (Fig. 19).

**Figure 20.**
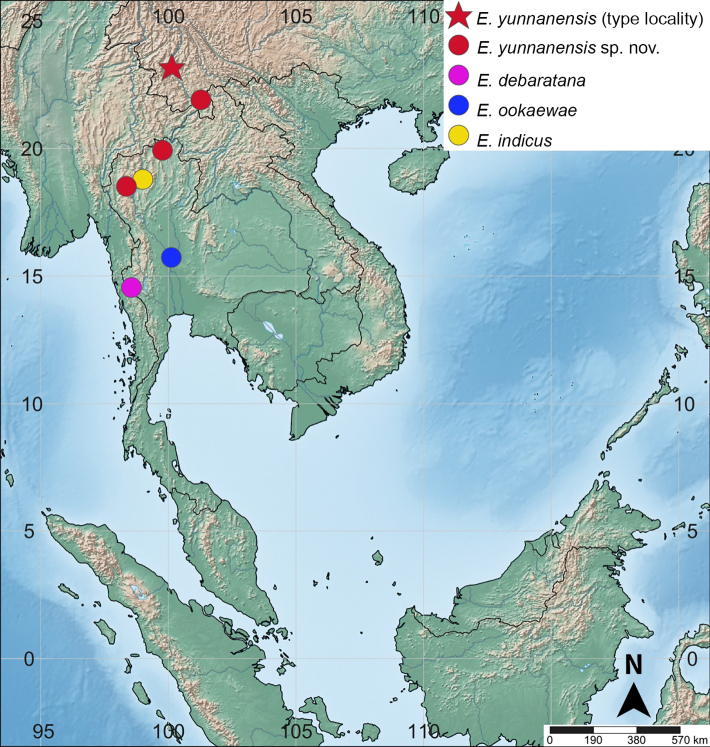
Distribution map of Ephoron yunnanensis sp. nov. and known Thai Ephoron species.

## Discussion

The present study addresses the long-standing taxonomic uncertainty of Ephoron yunnanensis by formally validating it, based on both morphological and molecular evidence. Previously treated as a nomen nudum, it has been hindered in recognition despite repeated mentions in the literature. The integration of specimens from both China and Thailand in this study provides robust support for its taxonomic status and expands its known distribution in tropical Asia.

Three valid species of Ephoron have been previously reported in Thailand: *E. indicus*, *E. ookaewae* and *E. debaratana* (Uéno 1961; Techakijvej et al. 2021; Kwanboon et al. 2025). In this study, Ephoron yunnanensis sp. nov. is formally described based on Chinese and Thai specimens. Our nymphal morphological evaluation of *E. yunnanensis* sp. nov., especially the frontal process and the number of tubercles on the tusk, revealed some similarity with *E. debaratana*. However, this species differs from *E. debaratana* in colouration and in the lack of pigment on the lateral branch trachea of the abdominal gill (Kwanboon et al. 2025). The study of Techakijvej et al. (2021) revealed the unique nymphal characters of *E. ookaewae* that are different from other Ephoron species, including mandibular tusks that are not curved inwards, relatively large, 5–7 tubercles on the tusk and a 3–4-pointed median frontal process. The egg structure of *E. yunnanensis* sp. nov. is characterised by a smooth chorion, which differs from that of other Asian species, such as *E. ookaewae* and *E. nanchangi* (Techakijvej et al. 2021; Pang et al. 2024). A comparison of the nymphal characteristics of Thai Ephoron species is shown in Table 3.

**Table 3.** Comparison of adult characteristics of Thai Ephoron species.

**Species**	***E. indicus***	***E. ookaewae***	***E. yunnanensis* sp. nov**.
Wing color	Hyaline	Hyaline	Hyaline
Intercalaries between CuA and CuP veins	6	4	6
Fork of MA	Distal to RS fork	Distal to RS fork	Distal to RS fork
Pterostigma crossveins	Numerous	Numerous	Numerous
Male foretarsi	5 segments, 1^st^ segment long	5 segments, 1^st^ segment very short	5 segments, 1^st^ segment very short
Female tarsomeres	Fore legs with 1 tarsomere	Fore legs with 5 tarsomeres, other legs with 3 tarsomeres	Fore legs with 4 tarsomeres, other legs with 3 tarsomeres
Egg structure	Oval, chorion smooth	Acorn-shaped, chorion sculptured in hexagonal netted pattern	Elongate-oval, chorion smooth
References	Chopra 1927	Techakijvej et al. 2021	This study

The record of *E. indicus* from Thailand should be treated with caution. Uéno (1961) reported this species from Thailand based on a single female imago. However, no photographs or detailed morphological description were provided. Therefore, the reliability of this identification remains uncertain. Since several Ephoron species have been subsequently described from the Oriental region, the possibility that the specimen reported by Uéno (1961) represents another species cannot be excluded. Further examination of additional material is required to clarify the taxonomic status of *E. indicus* in Thailand.

The first comprehensive molecular analysis of this genus from the Oriental region was conducted by Kwanboon et al. (2025). Our molecular analysis based on COI sequences provides strong support for species delimitation. The relatively low intraspecific genetic distance of 2.52% between Thai and Chinese populations of *E. yunnanensis* sp. nov. indicates conspecificity despite geographic separation, while the high interspecific distances (20.37–22.10%) clearly separate *E. yunnanensis* sp. nov. from other Thai species. The maximum-likelihood phylogeny and ASAP delimitation consistently recovered *E. yunnanensis* as a well-supported monophyletic lineage, which is the sister group of *E. ookaewae*.

The mass swarming behaviour observed in *E. yunnanensis* sp. nov. is consistent with reports of other Asian Ephoron species. Large-scale emergences attracted to light have been recorded in *E. nanchangi* in China, where swarms may accumulate on roads and bridges, disrupting traffic (Pang et al. 2024). A similar emergence pattern has also been reported for *E. ookaewae* in Thailand (Techakijvej et al. 2021), with synchronised swarming occurring shortly after sunset. These observations suggest that mass emergence and strong phototactic behaviour are common traits within the genus.

In terms of habitat, *E. yunnanensis* sp. nov. and other Asian Ephoron species are associated with medium-sized to large river systems, where nymphs inhabit sandy–muddy substrates (Pang et al. 2024; Ishiwata 1996). Similarly, *E. ookaewae* has been reported from large lowland rivers in Thailand (Techakijvej et al. 2021). In contrast, *E. debaratana* appears to occupy a different ecological niche, being recorded from headwater streams (Kwanboon et al. 2025). This difference suggests ecological differentiation among Thai Ephoron species, potentially reflecting habitat specialization along a river continuum gradient.

The first-instar nymph of *E. yunnanensis* sp. nov. described in this study shows general similarity to the early-instar morphology reported for *E. leukon* (O’Donnell 2009), particularly in its overall body plan, fully functional mouthparts, and limited development of the gill in the first instar. However, several differences are evident when compared with *E. leukon*. In *E. yunnanensis* sp. nov., the structure of the mouthparts, especially the mandibles and tusk, appears to be more distinctly developed than in *E. leukon*, as described in previous studies.

In this study, we validate *E. yunnanensis* sp. nov. using specimens from northern Thailand and southern China, combining morphological and molecular evidence. In addition, we provide a description of the first instar, offering new insights into the early developmental stages of *E. yunnanensis* sp. nov. These findings not only clarify the taxonomic status of the species but also expand its known distribution and contribute to a better understanding of the diversity within Ephoron in tropical Asia.

### Key to Thai Ephoron species of mature nymphs (based on Techakijvej et al. 2021)

**Table:** 

1	Gills on abdominal segments 2–7 with lateral tracheal branches pigmented	***E. debaratana***
–	Gills on abdominal segments 2–7 with lateral tracheal branches not pigmented (Fig. 6B)	**2**
2	Frontal process triangular (Fig. 1A)	***E. yunnanensis* sp. nov**.
–	Three to four-pointed median frontal process; Relatively large tubercles on mandibular tusk	***E. ookaewae***

### Key to Thai Ephoron species of male imago (Chopra 1927; Techakijvej et al. 2021))

**Table:** 

1	Penis lobes asymmetrical with forceps asymmetrical (one lobe dorsal, other ventral); genitalia complex	***E. indicus***
–	Penis lobes symmetrical; left and right lobes similar in shape and position	**2**
2	Penis lobes elongate, slender; apices rounded; moderately deep median cleft, six intercalaries between CuA1 and CuA2 (Fig. 9A)	***E. yunnanensis* sp. nov**.
–	Penis lobes straight and diverging laterally, robust; apices blunt; genitalia compact, four intercalaries between CuA1 and CuA2	***E. ookaewae***

### Key to Thai Ephoron species of female imago

**Table:** 

1	Foretarsi with one segment	***E. indicus***
–	Foretarsi with more than one segments	**2**
2	Foretarsi with four segments, chorion of egg smooth	***E. yunnanensis* sp. nov**.
–	Foretarsi with five segments, chorion of egg not smooth	***E. ookaewae***

## Supplementary Material

XML Treatment for Ephoron yunnanensis

## Additional information

### Artificial Intelligence (AI) use

The authors accept full responsibility for the content of the manuscript, including the disclosure of any use of AI.

No AI tools were used in the preparation of this manuscript.

### Author ORCIDs

S. Kwanboon https://orcid.org/0000-0002-3858-9522

X.-H.-Y. Zheng https://orcid.org/0000-0002-1520-2540

C.-F. Zhou https://orcid.org/0000-0001-8785-5228

B. Boonsoong https://orcid.org/0000-0002-8166-0021

### Data availability

All of the data that support the findings of this study are available in the main text.
